# Lessons Learned in the Delivery of Live Remote Group Weight Loss and Physical Activity Interventions for Older Adults

**DOI:** 10.1093/geroni/igab046.1088

**Published:** 2021-12-17

**Authors:** Jason Fanning, Barbara Nicklas

## Abstract

Social connection lies at the root of lasting health behavior change, and as such most effective interventions are built around social tools. Group leaders and peers provide education, and act as models of successful change and collaborators in addressing common barriers to behavioral adoption and maintenance. Unfortunately, many older adults do not have access to high quality group programs due to factors such as limited transport options, lack of local availability, or worries over personal safety. Importantly, developing effective, synchronous remote group programming is not as simple as delivering an in-person session via teleconference software. Instead, careful consideration must be paid to technology selection, fostering effective group communication, and developing confidence for use of remote intervention tools. This symposium provides key lessons learned from three group-based activity and weight loss interventions for older adults that focused on live, remote interaction. Jason Fanning will share lessons from the MORPH study, which paired remote group-mediated behavioral counseling with dietary weight loss and the accumulation of aerobic activity across the day. Christina Hugenschmidt will share her experiences adapting a group program involving improvisational dance or social gaming for remote delivery. Kushang Patel will present results from a mixed-methods study on the feasibility and acceptability of a remotely-delivered exercise program for older adults with knee osteoarthritis. Finally, Barbara Nicklas will place these experiences in the context of the development of exercise interventions for older adults over time, and highlighting vital next steps for ensuring more older adults have access to this important behavioral medicine.

